# Fellowships, Grants, & Awards

**Published:** 2006-07

## Institutional Patient-Oriented Career Development Programs in the Environmental Health Sciences (K12)

The National Institute of Environmental Health Sciences (NIEHS) is committed to the support and early career promotion of new researchers who will serve in leadership roles to promote the understanding of the impact of environmental exposures on human health. The need for additional researchers is particularly acute in the integration of the environmental health sciences with research in human disease. This RFA is intended to support the early career development of doctoral-level scientists who can make substantial contributions to the understanding of human health and disease by using environmental sciences to study the etiology, pathogenesis, progression, and epidemiology of human disease.

Concerns about the declining role of physician scientists in the research workforce began to emerge over 20 years ago, and since 1994, Institute of Medicine and National Research Council reports have recommended that the National Institutes of Health increase its commitment to the development of scientists who are prepared to engage in clinical research and who are conceptually prepared to engage in research designed to shorten the time to clinical application of important basic research. Emphasis has been placed on increasing the ranks of clinical researchers and, in particular, individuals who are engaged in patient-oriented research.

In 1995, NIH convened a committee to review the status of recruitment and training of future clinical researchers. The report of this panel, issued in 1997, recommended the support of clinical research programs aimed at medical students, such as combined degree programs (MD/PhD) programs for clinical researchers; ensuring that postdoctoral training grants include formal course work or degree programs in clinical research; development of new support mechanisms for young and mid-career clinical investigators; and taking steps to reduce clinical researchers’ educational debts.

In response to these reports, in 1999, the NIH introduced three new types of career development awards: The Mentored Patient-Oriented Career Development Award (K23); the Mid-Career Patient-Oriented Research Career Development Award (K24); and the Clinical Research Curriculum Award (K30) to provide institutions with the funds to develop or expand formal course work in areas related to clinical research.

The Clinical Research Curriculum Award (K30) is designed to support the development or improvement of core courses which provide in-depth instruction in the fundamental skills, methodology, theories, and conceptualizations necessary for the well-trained, clinical researcher. This includes formal course work in the design of clinical research projects, hypothesis development, biostatistics, epidemiology, and the legal, ethical, and regulatory issues related to clinical research. The program is complementary to the Mentored Clinical Investigator (K08, K23) programs since the funding is provided for curriculum development and not for the support of the participants in the program.

More recently, the importance of the cultivation and training of a sufficiently large clinical research workforce to facilitate bench-to-bedside research and to work in the interdisciplinary, team-oriented environments that characterize current research programs has been further emphasized and promoted by a number of activities within the NIH Roadmap to accelerate medical discoveries to improve health. Under the Re-engineering the Clinical Research Enterprise, the Multidisciplinary Clinical Research Career Development Programs Request for Applications was first announced in 2003 to support the early career development of clinical researchers from a variety of disciplines engaged in all types of clinical research. The basis of training is a core curriculum, such as those developed in the Clinical Research Curriculum Award.

At the same time the NIH was recognizing the need to encourage the support of greater numbers of clinical researchers, the NIEHS was seeking to define and enlarge the role of clinical research in its programs. A series of meetings was held in 1999 to try to define the issues and develop strategies for future directions. In addition to the issues addressed by the NIH as a whole, particular impediments to the development of the field of clinical environmental health sciences research were noted. First is the lack of a discrete population base from which to attract talented physician and other patient-oriented research trained scientists. Meeting attendees recognized that the environmental health sciences need to become closely intertwined with the stream of clinical training in a variety of departments and to be made attractive to physicians and other clinical researchers with a wide set of backgrounds. Second, it must be recognized that the research collaborations of a patient-oriented research scientist may not naturally coincide with the clinical home department. Third, the importance of strong mentorship implies that the development of junior scientists engaged in patient-oriented research cannot proceed in the absence of strong environmental medicine and environmental health science role models and research funding to support clinical projects for research training. Lastly, an impediment to attracting physicians and other patient-oriented research scientists to the environmental health sciences has been that in many cases research has focused on mechanism based effects of environmental exposures so that the contribution of an environmental exposure to what is seen clinically is not well delineated. Therefore, more research into the etiology, pathophysiological progression, epidemiology, or human biological link between an environmental exposure and a clinical decrement in function is needed as a basis for the development of environmental medicine.

The objective of this Institutional Patient-Oriented Career Development Program in the Environmental Health Sciences is to establish strong, multidisciplinary institution-based programs that promote the career development and career transition of doctoral level scientists in multidisciplinary team settings to design and direct projects in patient-oriented environmental health sciences research.

This specific K12 initiative supports an institutional patient-oriented career development program in the environmental health sciences and is intended to build upon training and career development programs initiated by the NIH Roadmap activities in Re-engineering the Clinical Research Enterprise, and to complement the refocusing of the research efforts of the training and research programs of the NIEHS to include a greater emphasis on integration of basic and clinical research with a focus on environmental exposures and unique scientific opportunities relevant to the mission of NIEHS.

Programs supported by this initiative will be expected to be characterized by: 1) a core of formal didactic core experiences in research methodology for conducting hypothesis based integrative research and specific courses in environmental health sciences; 2) a base of high-quality research in environmental health sciences focusing on a particular aspect of disease etiology, pathogenesis, progression, or epidemiology; 3) a pool of talented early career patient-oriented research scholars from a variety of disciplines who plan to pursue careers in the clinical environmental health sciences; and 4) a unifying seminar series or topics course, along with grand rounds presentations and journal club, which will instill in the clinical scholars an appreciation for integrative research and research approaches to problems of environmental exposures and human biology, human pathophysiology, and human disease.

The Institutional Patient-Oriented Research Career Development Program in the Environmental Health Sciences should be fully integrated with the clinical research infrastructure of the institutional setting. A medical school and an accredited graduate school must be co-primary participants in the application. Other clinically related institutions, such as schools of public health, pharmacy, nursing, etc., are encouraged to participate. Programs are expected to incorporate a multidisciplinary approach to the development of the program and cover a spectrum of scientific disciplines critical to innovative patient-oriented research in the environmental health sciences. This includes physicians trained in numerous clinical specialties and subspecialties, and individuals with doctoral degrees in disciplines such as human genetics, pharmacology, nursing, statistics, epidemiology, psychology, veterinary medicine, and engineering. Programs should be sufficiently flexible to allow either physicians to be trained in basic environmental sciences or those with a PhD in basic science to become proficient and redirect their careers to patient-oriented research problems in the environmental health sciences.

Applications responsive to this RFA will also be expected to integrate and build on other NIEHS supported resources at the institutional setting, such at training grants (T32) (http://www.niehs.nih.gov/dert/training/t32.htm), research grants, NIEHS Core Centers (P30) (http://grants.nih.gov/grants/guide/rfa-files/RFA-ES-05-008.html), NIEHS DISCOVER Centers (http://grants.nih.gov/grants/guide/rfa-files/RFA-ES-06-001.html), Children’s Centers (http://grants.nih.gov/grants/guide/rfa-files/RFA-ES-05-004.html), etc.

Programs must include didactic and practical training in the design, conduct and analysis of patient-oriented research along with an emphasis on methods and problems in clinical environmental health sciences research. Programs must propose to use a didactic core curriculum which is presented to all Clinical Research Scholars, such as those developed by the K30 program or the NIH Roadmap Multidisciplinary Clinical Research Career Development Program as well as courses in environmental sciences (toxicology, environmental medicine, environmental physiology, etc.)

Examples of the multidisciplinary core curriculum include: 1) clinical research methodology (including hypothesis generation, protocol design, etc.); 2) epidemiology; 3) biostatistics; 4) informatics; 5) ethical issues (with specific information on application to environmental health issues); 6) ensuring the safety of subjects (application to environmental health issues); 7) compliance with regulatory requirements for clinical research; 8) team building, leadership and management skills; 9) strategic, tactical, and negotiation skills; 10) grant writing and career development; 11) interactions with industry; 12) project/laboratory management.

This standard clinical research curriculum should be augmented with courses and other didactic experiences that are specifically designed to give the scholar a firm grounding in the environmental health sciences and a research focus on environmental exposures and the particular aspect of the disease etiology, pathogenesis, progression, or epidemiology which is central to the theme of the program.

The first and second year of the Clinical Scholar’s program should be equally divided between the core curriculum and a specific research project that uses environmental sciences to understand human disease. The third through the fifth year of the program should primarily focus the scholar’s time on their research project.

NIH defines human clinical research (http://grants1.nih.gov/grants/policy/hs/glossary.htm) as: 1) patient-oriented research; 2) epidemiologic and behavioral studies; and 3) outcomes research and health services research. Studies falling under Exemption 4 for human subjects research are not considered clinical by the NIH definition. Patient-oriented research is further defined as research conducted with human subjects (or on material of human origin such as tissues, specimens, and cognitive phenomena) for which an investigator (or colleague) directly interacts with human subjects. Patient-oriented research includes: 1) mechanisms of human disease; 2) therapeutic interventions; 3) clinical trials; or 4) development of new technologies. Excluded from this definition are *in vitro* studies that utilize human tissues that cannot be linked to a living individual.

In order to be responsive to this announcement, all of the clinical scholars supported by the program must be engaged in patient-oriented research. Programs which propose research training in only epidemiologic research, behavioral studies, and outcomes, or intervention research will not be considered responsive to this announcement and will not be considered for review or funding.

In order to provide a cohesive focus for the program and the scholars supported, the application must describe a plan to impart an understanding of current research in the environmental health sciences, particularly the clinical and integrative aspects and emphasizing the relative roles of environmental exposures, genetics, and other co-factors in the etiology of human diseases. This may include currently available coursework, plus a seminar series, a topics course, journal clubs, discussions of case studies, grand round presentations, etc. which are specifically developed for the participants in the program.

In addition, programs should propose a unifying core of career development opportunities specifically for environmental health patient-oriented research scholars which facilitate the interactions and exchange among scholars, mentors, and the program directors, and collaboration among scholars.

Research Projects of the Scholars supported by this program are expected to have a defined impact on the environmental health sciences and be responsive to the mission of the NIEHS, which is distinguished from that of other institutes by its focus on research programs seeking to use environmental sciences to understand the cause, mechanisms, moderation, or prevention of a human disease or disorder, or relevant pathophysiologic process. Scholars who are supported by this K12 Institutional Patient-Oriented Career Development Program in the Environmental Health Sciences will also be expected to pursue research projects involving the study of an NIEHS mission relevant environmental exposure, and to pursue projects which can evolve in Clinical Career Development Award applications (K08, K23, K01, K99/R00, or research grant applications (R01, R03, R21) within the defined mission area of the NIEHS. Examples of environmental agents relevant to the NIEHS mission include industrial chemicals or manufacturing byproducts, metals, pesticides, herbicides, air pollutants and other inhaled toxicants, particulates or fibers, fungal, bacterial or biologically derived toxins. Agents considered to be outside the mission of the NIEHS which would not be appropriate research areas for scholars supported by this program include, but are not limited to: alcohol, chemotherapeutic agents, ionizing radiation, smoking (except for second hand smoke in children), drugs of abuse, pharmaceuticals, and infectious or parasitic agents, except when these are disease cofactors to an environmental toxicant exposure to produce the biological effect. Studies using model compounds are only responsive when proposals to extend the research to a relevant compound are included in the protocols.

As part of the research career development experience, scholars should understand the relevance of the exposure paradigm to human exposure, and the biological and clinical rationale for the link between the exposure and the relevant human disease. Research projects of scholars should emphasize the translational and integrative aspects of the environmental health sciences.

Each Institutional Patient-Oriented Career Development Program in the Environmental Health Sciences application should include a Governance Committee composed of scientists from the sponsoring Institution who have clinical and environmental health science research expertise, and including the Program Director and Co-Director(s). The Committee may use institutional or outside consultants if needed. The Governance Committee is responsible for making recommendations regarding the appointment of scholars to the program, monitoring scholar progress and making recommendations to the Program Director regarding their continuation, evaluating ongoing research activities for merit and relevance to the program’s theme, and making recommendations for the addition or deletion of mentors from the program. The Governance Committee is a formal part of the structure of the program. It should meet regularly and keep written minutes, which will be reviewed as part of a competing or noncompeting application. In addition, an annual evaluation is recommended.

Each Environmental Health Clinical Scholar appointed to the K12 award must be supervised by a mentoring team of at least two mentors from two different disciplines. One should have research expertise in the environmental exposure proposed in the research project and one should provide expertise in the clinical and patient-oriented research. Either through interactions with the mentoring team, or other career development activities, the mentoring team should insure the clinical scholar has appropriate scientific training in the basic and mechanistic scientific foundation for the clinical research problem under investigation.

The purpose of the K12 is to provide systematic support for the transition of clinical scientists from trainee to new mentored faculty. Therefore, how this transition is to be accomplished and the progress of the scholars monitored should be addressed in the application. Benchmarks for progress of the scholars should be outlined in the application.

Within the application, applicants must present a recruitment plan. The application should describe the potential pool of scholars, including the types of prior clinical and research training. The criteria to be used for candidate evaluation and selection should be described. In addition a plan for recruiting scholars with economically, socially, or culturally disadvantaged backgrounds, individuals with disabilities or from racial or ethnic groups that are currently underrepresented in biomedical, behavioral, or clinical sciences should be included in the recruitment plan described in the application.

Applicants should also describe a comprehensive evaluation and tracking component that will review the effectiveness of all aspects of the program (including scholars, courses, mentors, co-directors, mentoring effectiveness and institutional characteristics, and a system for tracking graduates throughout their career to determine the success rate of applying for and obtaining research support, and publications.

This funding opportunity will use the NIH Mentored Clinical Scientist Development Program (K12) institutional award mechanism.

As an applicant, you will be solely responsible for planning, directing, and executing the proposed program.

This funding opportunity uses the just-in-time budget concepts. It also uses the nonmodular budget format described in the PHS 398 application instructions (see http://grants.nih.gov/grants/funding/phs398/phs398.html). A detailed categorical budget for the Initial Budget Period and the Entire Proposed Period of Support is to be submitted with the application PHS 398 application instructions.

The PHS 398 application instructions are available at http://grants.nih.gov/grants/funding/phs398/phs398.html in an interactive format. Applicants must use the currently approved version of the PHS 398. For further assistance contact GrantsInfo, 301-435-0714 (telecommunications for the hearing impaired: TTY 301-451-0088) or by e-mail: GrantsInfo@nih.gov.

Applications must be prepared using the current PHS 398 research grant application instructions and forms. Applications must have a D&B Data Universal Numbering System (DUNS) number as the universal identifier when applying for Federal grants or cooperative agreements. The D&B number can be obtained by calling 866-705-5711 or through the web site at http://www.dnb.com/us/. The D&B number should be entered on line 11 of the face page of the PHS 398 form.

The letter of intent receipt date for this RFA is September 23, 2006, with the application of receipt date October 23, 2006. The complete version of this RFA is available at http://grants.nih.gov/grants/guide/rfa-files/RFA-ES-06-005.html.

Contact: Carol Shreffler, Division of Extramural Research, National Institute of Environmental Health Sciences, EC-23, Building 4401, Room 3411, P.O. Box 12233, Research Triangle Park, NC 27709 USA, 919-541-1445, fax: 919-541-5064, e-mail: shreffl1@niehs.nih.gov.

Reference: RFA-ES-06-005.

